# Current chemoprevention approaches in Lynch syndrome and Familial adenomatous polyposis: a global clinical practice survey

**DOI:** 10.3389/fonc.2023.1141810

**Published:** 2023-05-24

**Authors:** Kathryn A. Mraz, Rachel Hodan, Linda Rodgers-Fouche, Sanjeevani Arora, Francesc Balaguer, Jose G. Guillem, Joanne M. Jeter, Priyanka Kanth, Dan Li, David Liska, Joshua Melson, Kimberly Perez, Charite Ricker, Brian H. Shirts, Eduardo Vilar, Bryson W. Katona, Mev Dominguez-Valentin

**Affiliations:** ^1^ Department of Genetics, Grey Genetics, Brooklyn, NY, United States; ^2^ Research Department, Center for Genomic Interpretation, Sandy, UT, United States; ^3^ Cancer Genetics, Stanford Health Care, Palo Alto, CA, United States; ^4^ Center for Cancer Risk Assessment, Massachusetts General Hospital, Boston, MA, United States; ^5^ Cancer Prevention and Control Program, Fox Chase Cancer Center, Philadelphia, PA, United States; ^6^ Department of Gastroenterology, Hospital Clínic de Barcelona, Institut d’Investigacions Biomèdiques August Pi i Sunyer (IDIBAPS), Centro de Investigación Biomédica en Red de Enfermedades Hepáticas y Digestivas (CIBEREHD), University of Barcelona, Barcelona, Spain; ^7^ Division of Gastrointestinal Surgery, University of North Carolina-Chapel Hill, Chapel Hill, NC, United States; ^8^ Department of Internal Medicine, Huntsman Cancer Institute, University of Utah, Salt Lake City, UT, United States; ^9^ Department of Gastroenterology, MedStar Georgetown University Hospital, Washington, DC, United States; ^10^ Department of Gastroenterology, Kaiser Permanente Medical Center, Santa Clara, CA, United States; ^11^ Department of Colorectal Surgery and Sanford R. Weiss MD Center for Hereditary Colorectal Neoplasia, Digestive Disease and Surgery Institute, Cleveland Clinic, Cleveland, OH, United States; ^12^ Division of Gastroenterology, University of Arizona Cancer Center, Tucson, AZ, United States; ^13^ Department of Medical Oncology, Dana-Farber Cancer Institute/Harvard Medical School, Boston, MA, United States; ^14^ Division of Medical Oncology, Department of Medicine, Keck School of Medicine, University of Southern California, Los Angeles, CA, United States; ^15^ Department of Laboratory Medicine and Pathology, University of Washington, Seattle, WA, United States; ^16^ Department of Clinical Cancer Prevention, The University of Texas MD Anderson Cancer Center, Houston, TX, United States; ^17^ Division of Gastroenterology and Hepatology, University of Pennsylvania Perelman School of Medicine, Philadelphia, PA, United States; ^18^ Department of Tumor Biology, Institute of Cancer Research, The Norwegian Radium Hospital, Oslo, Norway

**Keywords:** chemoprevention, CRC, Lynch syndrome, Familial adenomatous polyposis, FAP, barriers, aspirin

## Abstract

**Background:**

International chemoprevention preferences and approaches in Lynch syndrome (LS) and *APC-*associated polyposis, including Familial adenomatous polyposis (FAP) and attenuated FAP (AFAP) have not been previously explored.

**Aim:**

To describe current chemoprevention strategies for patients with LS or FAP/AFAP (referred to collectively as FAP) practiced by members of four international hereditary cancer societies through administration of a survey.

**Results:**

Ninety-six participants across four hereditary gastrointestinal cancer societies responded to the survey. Most respondents (91%, 87/96) completed information regarding their demographics and practice characteristics relating to hereditary gastrointestinal cancer and chemoprevention clinical practices. Sixty-nine percent (60/87) of respondents offer chemoprevention for FAP and/or LS as a part of their practice. Of the 75% (72/96) of survey respondents who were eligible to answer practice-based clinical vignettes based off of their responses to ten barrier questions regarding chemoprevention, 88% (63/72) of those participants completed at least one case vignette question to further characterize chemoprevention practices in FAP and/or LS. In FAP, 51% (32/63) would offer chemoprevention for rectal polyposis, with sulindac - 300 mg (18%, 10/56) and aspirin (16%, 9/56) being the most frequently selected options. In LS, 93% (55/59) of professionals discuss chemoprevention and 59% (35/59) frequently recommend chemoprevention. Close to half of the respondents (47%, 26/55) would recommend beginning aspirin at time of commencement of the patient’s first screening colonoscopy (usually at age 25yrs). Ninety-four percent (47/50) of respondents would consider a patient’s diagnosis of LS as an influential factor for aspirin use. There was no consensus on the dose of aspirin (≤100 mg, >100 mg - 325 mg or 600 mg) to offer patients with LS and there was no agreement on how other factors, such as BMI, hypertension, family history of colorectal cancer, and family history of heart disease, would affect the recommendation for aspirin use. Possible harm among older patients (>70 years) was identified as the most common reason to discourage aspirin use.

**Conclusion:**

Although chemoprevention is widely discussed and offered to patients with FAP and LS by an international group of hereditary gastrointestinal cancer experts, there is significant heterogeneity in how it is applied in clinical practice.

## Background

Colorectal cancer (CRC) is the third most common cancer worldwide (1). In 2020, there were over 1,931,590 new cases of CRC and 935,173 people died of the disease (1). Although most CRC cases are sporadic, 5-10% are due to hereditary syndromes, the two most common of which are Lynch syndrome (LS) and *APC*-associated polyposis (including Familial adenomatous polyposis (FAP) and attenuated familial adenomatous polyposis (AFAP)), collectively referred to here as FAP (2–4). LS is an autosomal dominant condition which affects approximately 1 in 300 individuals and is caused by pathogenic germline variants in the mismatch repair (MMR) genes or a 3’ deletion of *EPCAM* (5). The lifetime risk of developing CRC varies widely between individuals within a LS family and also among distinct LS families, ranging from 30% to 80% (6, 7). FAP is an autosomal dominant condition caused by pathogenic germline variants in the *APC* gene and is characterized by the development of hundreds to thousands of colon adenomas (2). This syndrome represents up to 1% of all CRC cases, but carriers of *APC* pathogenic variants have an almost 100% lifetime risk of developing CRC in the absence of surveillance and/or risk-reducing surgery (2).

Chemoprevention strategies have been reported to reduce CRC incidence and mortality and their use has been recommended for average-risk as well as high-risk groups (8). CRC chemoprevention agents include aspirin, non-aspirin nonsteroidal anti-inflammatory drugs (NA-NSAIDs), statins, agents that target metabolic pathways, vitamins, and minerals. Daily aspirin intake (600 mg per day) has been shown to reduce the risk of CRC in LS (9). However, there is limited awareness of LS and the preventive effects of aspirin among general practitioners (10). The NA-NSAID sulindac has been described to temporarily control the growth of colon adenomas in FAP patients and significantly decrease the formation of aberrant crypt foci (ACF), a precursor to colon adenomas and cancers (11, 12). Nair et al. found that a sulindac dose of 30mg/kg/day also resulted in polyp reduction (13) and was equivalent to half the dosage in the primary sulindac trial focusing on FAP patients who were given 150 mg of sulindac twice daily (14). Aspirin, which has the ability to irreversibly inhibit both COX-1 and COX-2 isoenzymes (15), was evaluated in the Colorectal Adenoma/Carcinoma Prevention Programme (CAPP) 1 study. CAPP1 evaluated the impact of aspirin-600 mg/d with or without resistant start at 30 g/d vs. placebo on the number of rectosigmoid polyps in an international Randomized clinical trial (RCT) in children and young adults spanning from 10-21 years of age, finding aspirin decreased polyp size, but overall polyp burden did not change, suggesting a role of aspirin in slowing disease vs. hindering its initiation (16). In a recent multicenter, double-blind randomized, two by-two factorial design trial of patients with FAP completed by Ishikawa et al, low- dose aspirin was found to safely suppress recurrent colorectal polyps larger than 5 mm (17). Additionally, the enteric-coated formulation of omega-3 polyunsaturated fatty acid eicosapentaenoic acid (EPA) and selective cyclo-oxygenase-2 inhibitors (18) have also been reported to have chemopreventive effects in FAP patients, with similar efficacy in risk reduction.

Multiple gastroenterology and oncology professional societies across North America, Europe and Australia all address chemoprevention recommendations with regard to LS and/or FAP, most of which recommend chemoprevention should be considered in patients with LS, though FAP is less commonly addressed (**Supplementary Table 1S**). Furthermore, guidelines often do not provide detailed guidance on determining which patients will benefit most, nor provide specifics regarding initiation age or dosing (19).

Given the lack of uniform recommendations addressing use of chemoprevention in FAP and LS, we investigated current trends in chemoprevention practice strategies for patients with FAP and LS by surveying members of four international hereditary gastrointestinal cancer societies.

## Methods

### Study population

The Collaborative Group of the Americas on Inherited Gastrointestinal Cancer (CGA-IGC) collaborated with three international hereditary cancer societies (Latin America Hereditary Tumor Group (LA-GETH), International Society for Gastrointestinal Hereditary Tumours (InSiGHT) and the European Hereditary Tumor Group (EHTG) to reach healthcare providers who manage patients with FAP and LS. This is the first effort to report on current FAP and LS chemoprevention strategies on a global level and not country specific.

### Survey structure

A 33-item survey, including Likert scoring, was designed to capture whether chemoprevention is offered/considered for patients with FAP and LS; when chemoprevention is prescribed and which type/dosage, the age groups considered for chemoprevention (based on four clinical cases) and to determine barriers for chemoprevention practice (**Supplementary Survey**). Respondents who indicated they were members of more than one society and who had previously taken the survey were redirected to the end of the survey (after completing the survey once) to disallow multiple responses from a single respondent. Two FAP and two LS case vignettes were presented to gauge the current practices of care management with a specific focus on chemoprevention recommendations of providers. If the respondents selected “Strongly agree” AND/OR “Uncertain” to *all* of the barrier questions, the participants were routed to bypass the case vignette portion of the survey and redirect to the end of the survey. The case vignettes were skipped for these respondents that had already indicated to the chemoprevention barrier questions that either A) *ALL* of the barriers regarding chemoprevention applied to them in their practice *and/or* B) they were Uncertain regarding the barriers regarding chemoprevention and considered in-depth chemoprevention knowledge to not be within the purview of their services (**Supplementary Survey**). Each case vignette had several questions with skip logistic such that an individual’s response to the first question would determine the next question administered or send them to the subsequent case vignette. The questions regarding colon management modality (Case 1 and Case 3), chemoprevention options (Case 1) and chemoprevention dosage (Case 3) were retroactively regrouped based on the responses (detailed in **Supplementary Tables 2S–4S**). Specifically, the management and chemoprevention questions were “select all” which resulted in a split of respondents who selected only one option and a group of respondents who selected more than one option, which is reflected in the data (**Supplementary Survey**; **Supplementary Tables 2S, 2.1S, 2.2S, 3S**). We inquired about chemoprevention dosage in LS: 100 mg, 300 mg, and 600 mg of aspirin in addition to an “Other” option with free text to allow entries of additional dosing. Data review of 17 write-in responses allowed for the following revised categories: ≤100mg, >100 mg – 325 mg, 600 mg. The original responses are available in **Supplementary Table 4S**.

The survey was administered electronically *via* SurveyMonkey between November 2021 and March 2022 with a link sent to all members of the four participating international hereditary cancer societies. Survey responses were anonymized. Some questions had a notable number of write-in responses or select all responses which lead to a retroactive regrouping of data.

### Ethical approval

The study was reviewed and granted an exemption by the Regional Committee for Medical & Health Research Ethics, Section D, South East Norway. The research project was assessed in accordance with the Norwegian Research Ethics Act 2006 and Act on Medical and Health Research 2008.

## Results

### Survey response

**Figure 1:**
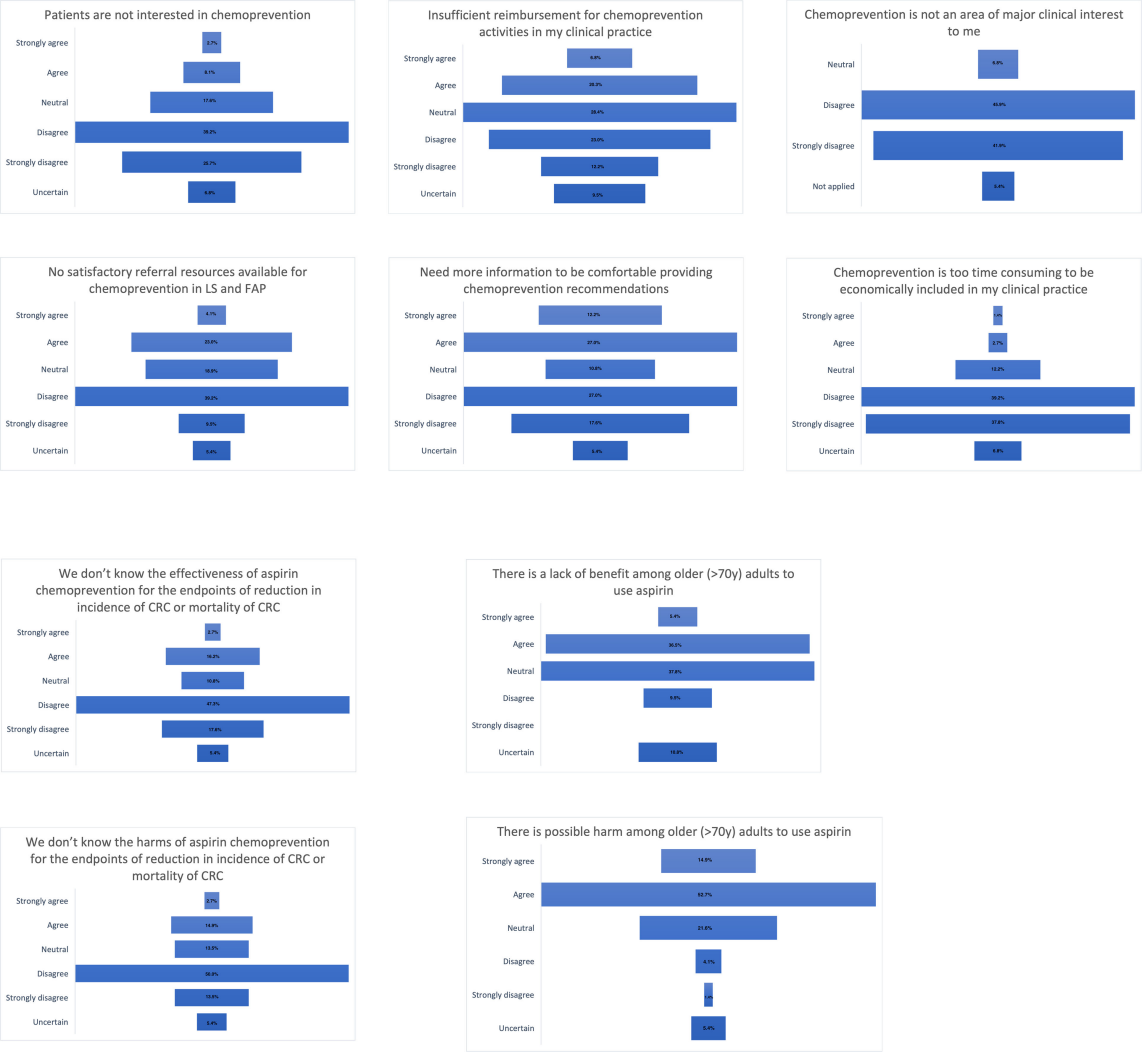
Clinical practice setting barriers for CRC chemoprevention practice, N=74.

Ninety-six respondents completed at least some part of the survey, with 91% (87/96) completing the demographics and practice characteristics relating to hereditary gastrointestinal cancer and chemoprevention clinical practice (**Table 1**), 77% (74/96) responding to the questions pertaining to barriers for chemoprevention in practice (**Figure 1**), and 66% (63/96) providing responses addressed in the clinical cases tables. Half of the respondents belonged to CGA-IGC (51%, 49/96), followed by InSiGHT (27%, 26/96), LA-GETH (16%, 15/96) and EHTG (6%, 6/96).

**Table 1:** Demographics and practice characteristics of clinician respondents.

Demographics characteristics	n	%
Practice location		
Academic Medical Center with patient care practice	72	75.0
Community Hospital based practice	9	9.4
Private patient care practice	8	8.3
Academic Medical Center without patient care practice	3	3.1
Other	4	4.2
Specialty		
Gastroenterologist	34	35.4
Colorectal surgeon	15	15.6
Genetic Counselor in Cancer Genetics	13	13.5
Medical geneticist	10	10.4
Medical oncologist	9	9.4
Other	15	15.6
Geographic location		
North America	50	52.1
USA	45	
Canada	5	
Australia/New Zealand	4	4.2
South America	15	15.6
Europe	16	16.7
Asia	4	4.2
United Kingdom	7	7.3
Practice characteristics		
Years providing hereditary gastrointestinal cancer risk assessment		
Less than 5 years	12	13.8
5-10 years	19	21.8
10 years or more	56	64.4
Number of LS and FAP patients seen per week		
Less than 5	48	55.2
5-10	27	31.0
More than 10	10	11.5
NA	2	2.3
Offer chemoprevention for LS and/or FAP patients as a part of their practice		
Yes	60	69.0
No	20	23.0
Other	7	8.0
Years that their hospital/service started prescribing chemoprevention		
1989-2009	3	6.0
2010-2021	28	56.0
Uncertain	19	38.0

### Demographics and practice characteristics

**Table 1** shows the demographics and practice characteristics of the respondents. Most of the respondents work in a patient care practice within an academic medical center (75%) and 35% (34/96) were gastroenterologists. Most respondents work in North America (52%) followed by Europe (17%) and South America (16%). More than half (64%) of respondents reported 10 years or more of experience in clinical practice, and most (86%) see between 1-10 FAP or LS patients per week. Sixty-nine percent of the respondents offered chemoprevention to patients with FAP and/or LS as a part of their practice and 56% began using chemoprevention in 2010 or later.

Seventy-five percent (72/96) of respondents were eligible to provide responses to the case vignettes. Eighty-eight percent (63/72) of respondents who entered into the case vignette section of the survey completed at least one of the case-based questions for analysis.

### Barriers in practice

**Figure 1** describes the responses for the 10 questions addressing clinical practice barriers. Of the responses (n=74), 65% (48/74) reported that patients are interested in chemoprevention and half (49%) indicated there were satisfactory referral resources available. There was no agreement if there is sufficient reimbursement for chemoprevention activities in clinical practice. Forty percent of respondents endorsed a need for more data in order to feel comfortable providing cancer prevention recommendations. Chemoprevention was widely agreed upon being an area of major clinical interest (88%) and most respondents agreed there is enough clinical time for discussion (77%). The effectiveness and harm of aspirin chemoprevention was known by most of the respondents (65% and 64%, respectively). Less than half of the respondents (42%, 31/74) agreed that there is a lack of benefit among older people but most of them (68%, 50/74) agreed that there is a possible harm among older people to use aspirin.

### Chemoprevention strategies in FAP (Case 1 and 2)

**Figure 2:**
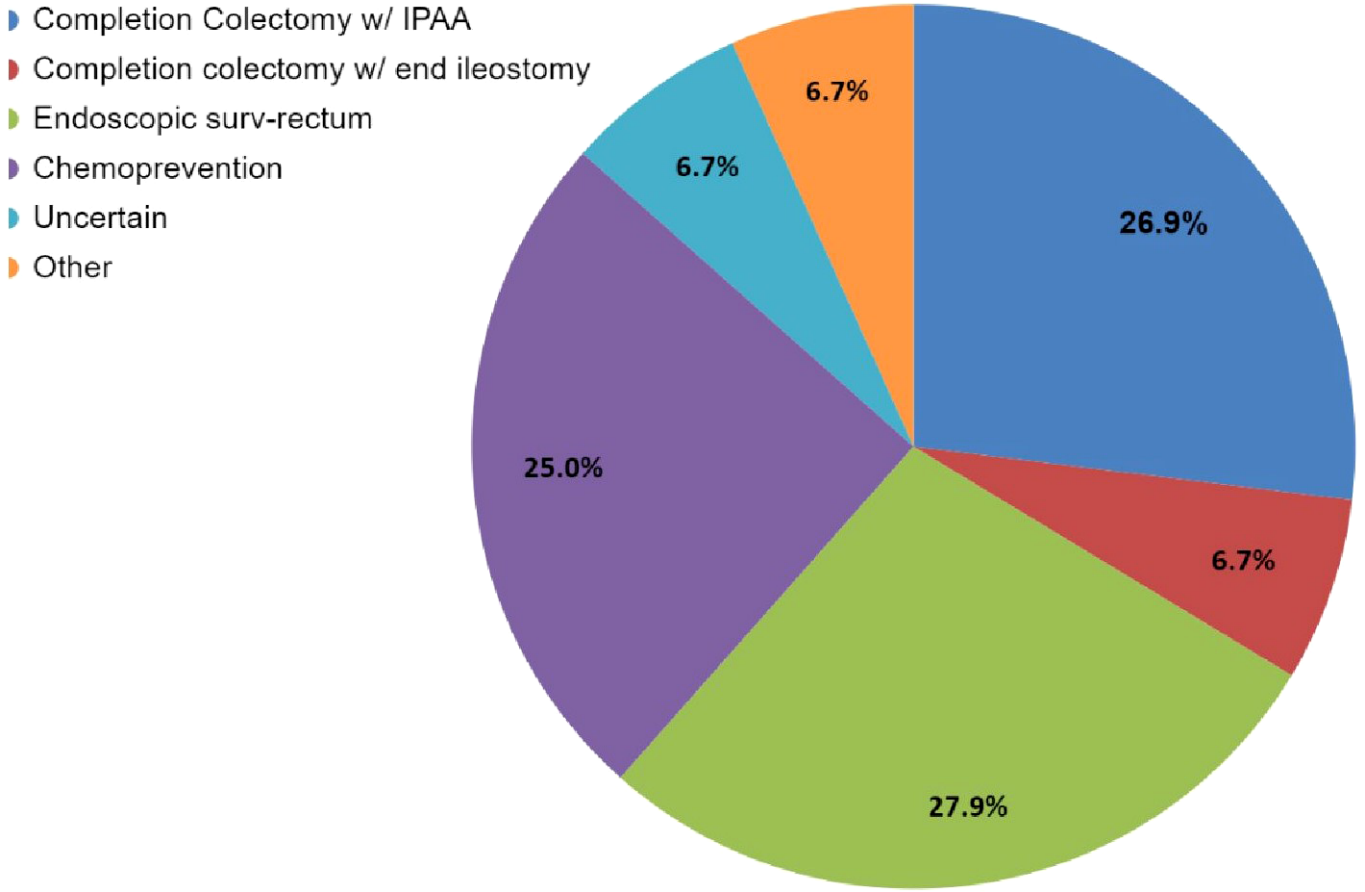
Case 1 – Rectal polyp management options selected by 63 respondents. The management question for Case 1 allowed participants to select more than one option. While this image depicts the cumulative number of selections for each option, it does not show the combinations that were selected. Refer to **Supplementary Tables 2S, 2.1S** for specific combinations of management. Respondents who selected “Other, specify” (n=7) had the option for a write-in option; this could *either* be further elaborate on their modality selection answers and/or to provide an answer that was not given as an option; 3/7 answers centered around chemoprevention not being in the purview of the specialist taking the survey (and wanting to consult with other specialists) (which also selected ‘Uncertain’); while the other 4/7 (who also selected one or multiple management modalities) described in which order the modalities selected would take place and the thought process in their workup for in their evaluations, and their reasoning, and preference for wanting to know optimal dosing. Refer to **Supplementary Table 2.1S** for detailed write in responses. Of note “Endoscopic surv-rectum” in the legend = endoscopic surveillance of the rectum.

**Figure 3:**
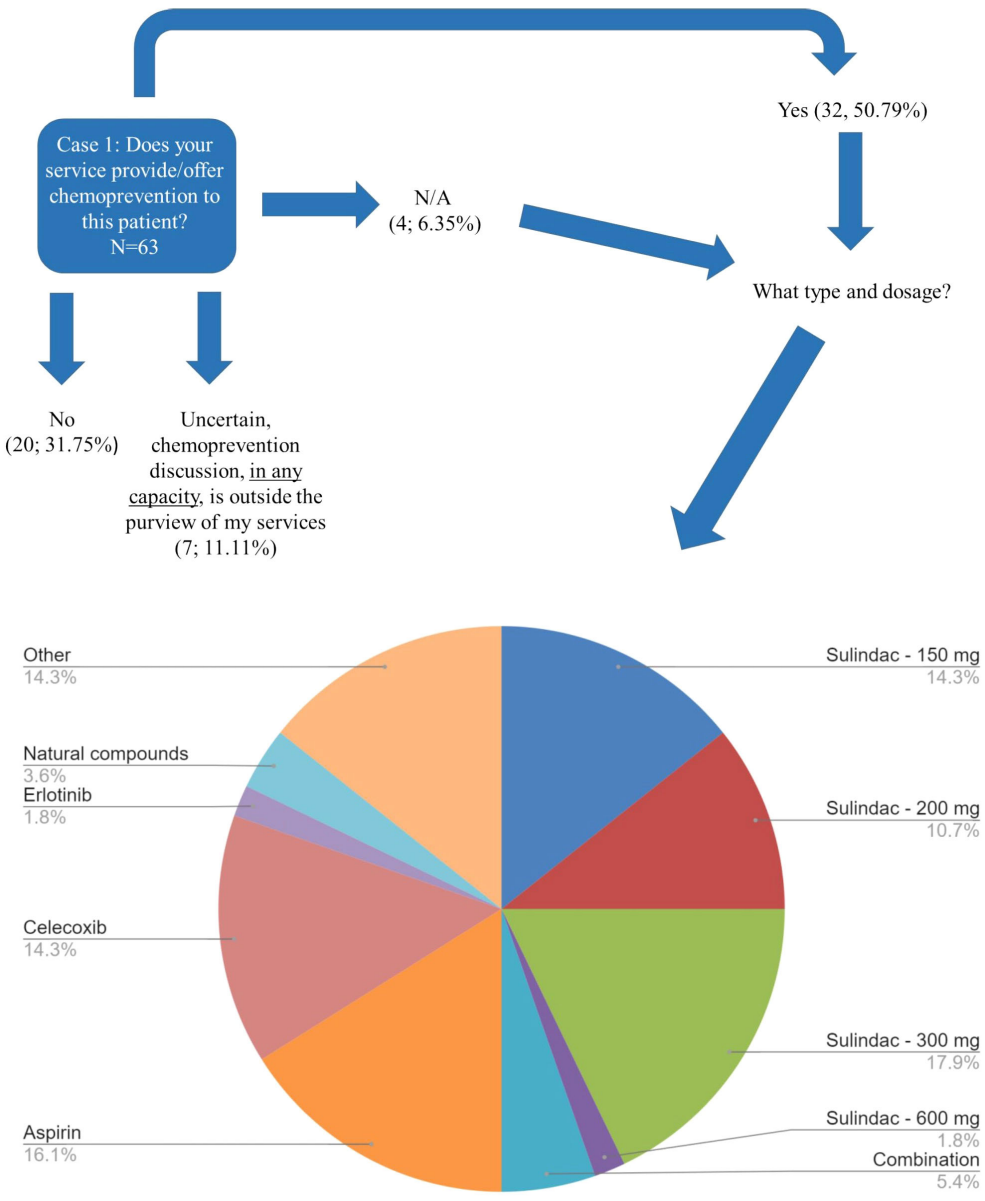
Case 1 Type of Chemoprevention recommended - FAP. Responses of 63 participants regarding their recommendation regarding whether chemoprevention would be offered by their service to the patient affected with FAP described in Case 1. A subset of respondents (n=35) selected the specific type/dosage of chemoprevention offered. Respondents were allowed to select more than one option. This image depicts the 56 total selections of each management type. Of note, "Combination" is difluoromethylornithine (DFMO) plus sulindac, dosage unknown; Other: respondents could write in a response by selecting “Other”. Other could be to clarify their selection(s) and/or to provide an additional response that was not previously provided as an option. The most common write-in response was “clinical trials”. Refer to **Supplementary Table 2.2S** for the combinations that were selected.

Case 1 described a 33-year-old male patient with FAP with a sub-total colectomy with an ileorectal anastomosis, who presented with significant rectal and duodenal polyposis in addition to a desmoid tumor (**Supplementary Survey**). Of the 63 respondents, 44% (27/63) selected more than one management modality for the patient’s rectal polyposis presented in Case 1 (**Supplementary Table 2S**). When the 103 total selections of the 63 respondents were reviewed, three modalities were found to be common recommendations: endoscopic surveillance of the rectum (28%, 29/103) followed closely by completion colectomy with ileal pouch anal anastomosis (IPAA) (27%, 28/103) and then chemoprevention (25%, 26/103) (**Figure 2**; **Supplementary Tables 2S**, **2.1S**). Respondents were more likely to consider completion colectomy with IPAA (19/28) as a sole management approach compared to other options as depicted in **Supplementary Tables 2S**, **2.1S**. The most common management approach that included ≥1 management modality was chemoprevention AND endoscopic surveillance of the rectum (57%, 16/28). Over half of respondents (51%, 32/63) would offer chemoprevention to Case 1 (**Figure 3**) with the most common types and doses of chemoprevention agents being: sulindac-300mg (18%) and aspirin (16%) as detailed further in **Figure 3**. Respondents had the option to select multiple chemoprevention options to manage Case 1’s rectal polyposis (**Figure 3**; **Supplementary Table 2.2S**). Among the 35 respondents, 23 selected only one chemoprevention modality, the most common of which was aspirin (25%, 6/23). Over one third (12/35) of those who selected a chemoprevention agent for Case 1 indicated offering >1 chemoprevention, each combination was unique except for one combination of Sulindac-300mg + Celecoxib which was selected by two respondents.

Case 2 described a patient with a clinical diagnosis of FAP and worsening polyp burden in his teenage years (**Supplementary Survey**). We inquired whether chemoprevention would be offered to the patient at 15 years of age. Of the 59 respondents, 92% (54/59) who indicated chemoprevention is within their purview, 30% (16/54) would provide/offer chemoprevention to the described patient with FAP (**Table 2**). Of the 23 respondents who continued this vignette based on the survey design (**Supplementary Survey**) focusing on specific patient age ranges for FAP chemoprevention, 35% (8/23) indicated they offer chemoprevention to a specific age group of FAP patients; 35% (8/23) do not offer it to a specific age group, and the remaining 6/23 were uncertain. Of the 65% (15/23) of respondents who answered additional questions regarding age specifications detailed in **Table 2**, 53% (8/15) recommended the 18y-25y age range to begin chemoprevention in FAP.

**Table 2:** Case 2 Current chemoprevention practice for pediatric patients with FAP.

Case 2 Inquiries:		
Does your service provide/offer chemoprevention to this patient?		
	n=59	
	n	%
Yes	16	27.1
No	34	57.6
Uncertain, chemoprevention discussion, **in any capacity,** is outside the purview of my services	5	8.5
N/A	4	6.8
Is chemoprevention offered to specific age group for your FAP patients?		
	n=15	
	n	%
Yes	8	34.8
No	8	34.8
Uncertain	6	26.1
N/A	1	4.4
Which age group (years)?		
	n=15	
	n	%
<18y	2	13.3
18y-25y	8	53.3
26y-35y	1	6.7
36y-45y	1	6.7
>45y	1	6.7
Other	2	13.3

### Chemoprevention strategies in LS (Case 3)

Case 3 described a 45-year-old female patient with LS due to a pathogenic *MSH2* variant of maternal inheritance and a history of metachronous CRC at age 27 and 32. Although the patient had prior CRC diagnoses, she had much of her remaining colon intact (**Supplementary Survey**). Of the 59 respondents, 73% (43/59) selected more than one modality to manage the patient’s CRC risk (**Supplementary Table 3S**). The most common (88%, 38/43) multi-modality selected was chemoprevention AND endoscopic surveillance. Among the responses that selected only one management option (27%, 16/59), endoscopic surveillance was the most common (38%, 6/16).

**Figure 4:**
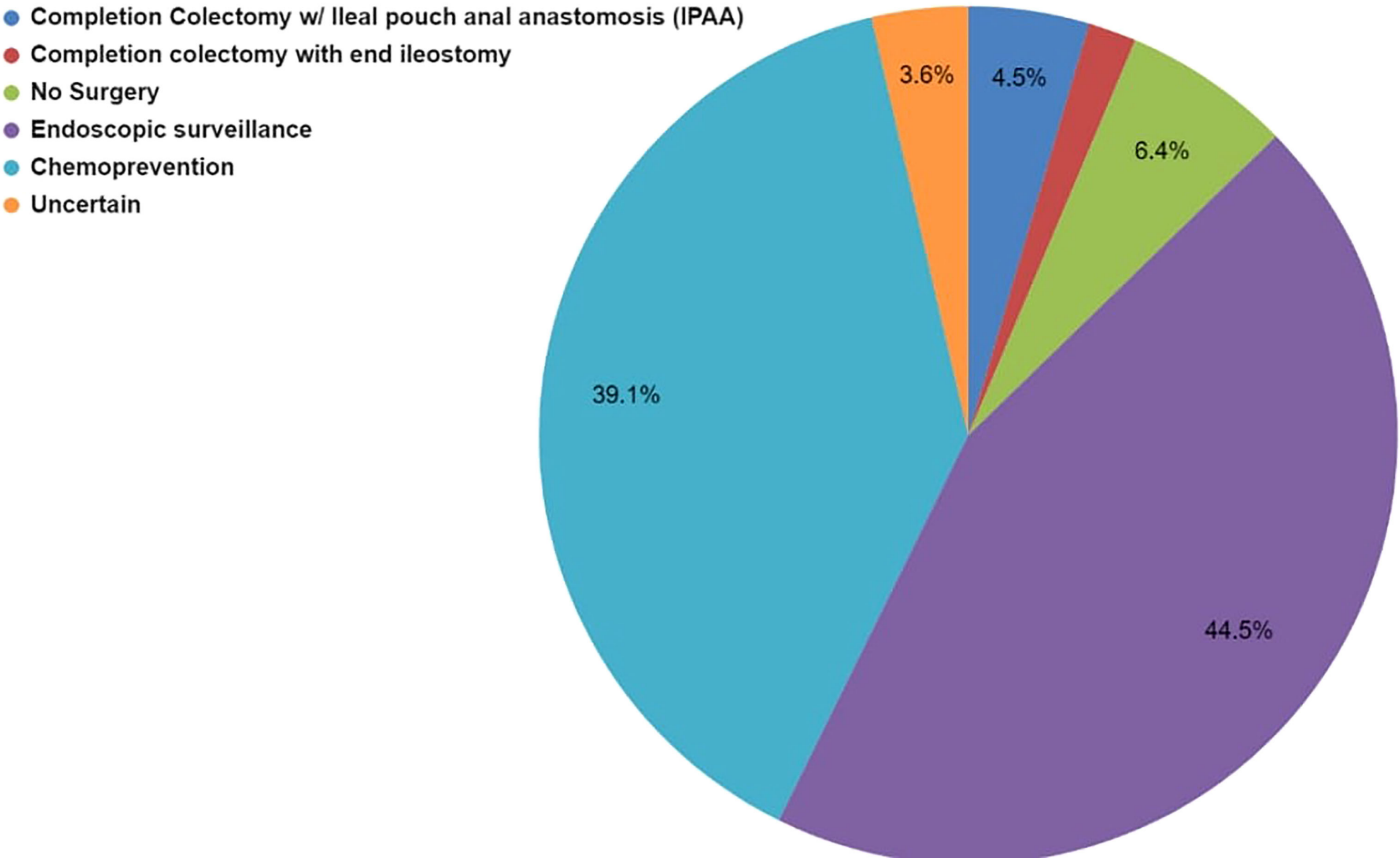
Case 3 - Collective management options to manage the LS patient’s rectal polyps selected by 59 respondents for Case 3. Respondents were allowed to select more than one option. While this image depicts the cumulative number of selections for each management type, it does not show the combinations that were selected. Respondents selected either one or multiple management modalities, selections are further detailed in **Supplementary Table 3S**.

**Figure 5:**
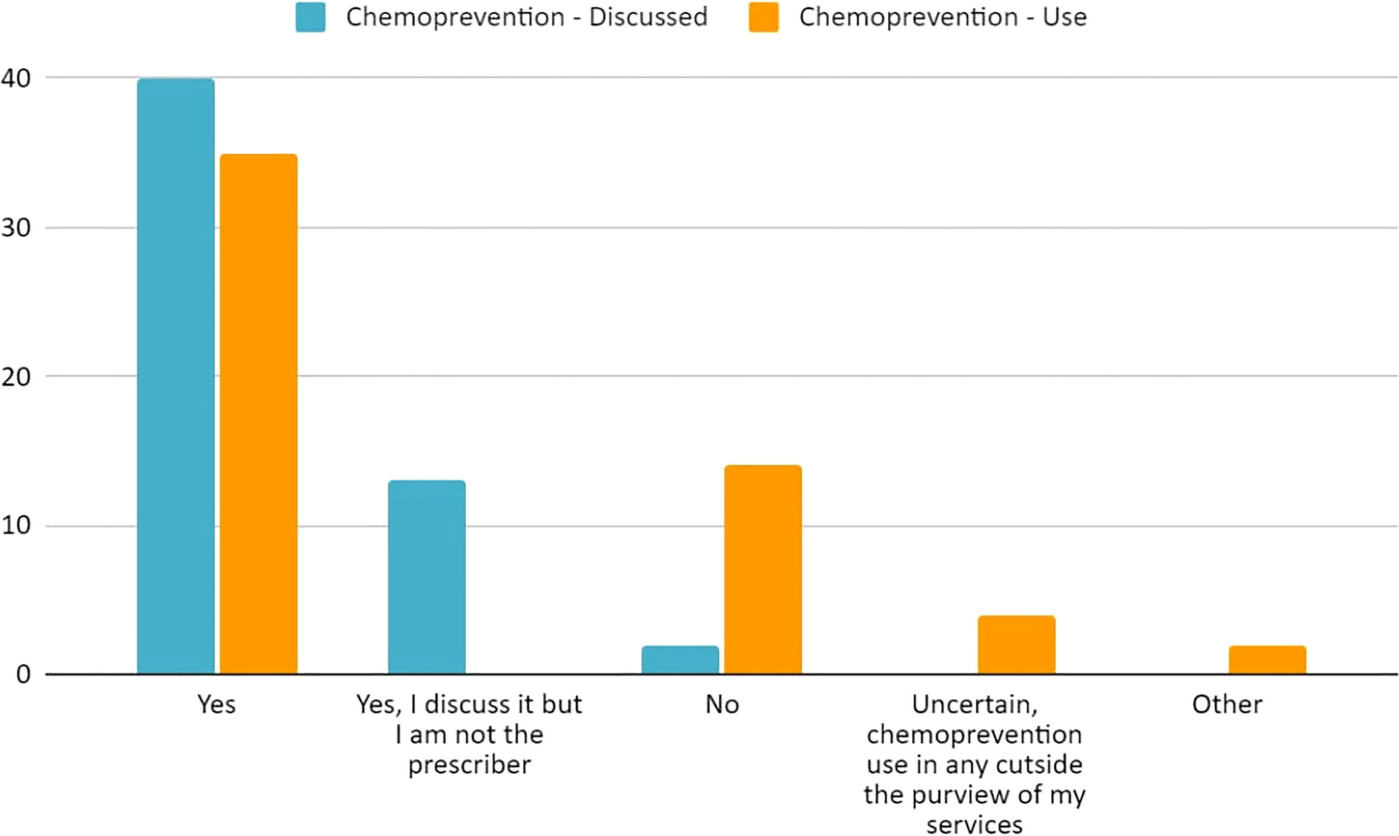
Current practices of 59 respondents regarding chemoprevention discussion and use regarding our populations’ patients with LS.

Overall, the most common management modality was endoscopic surveillance (43%, 49/113) followed closely by chemoprevention (38%, 43/113) (**Figure 4**; **Supplementary Table 3S**). Fifty-nine percent (35/59) of the respondents across the four hereditary cancer societies characterize chemoprevention as *frequently used* in patients with LS (**Table 3**). Most of the respondents discuss (93%, 55/59) and use (59%, 35/59) chemoprevention with their patients with LS (**Table 3** and **Figure 5**). We inquired about specific aspirin dosage for chemoprevention. Nearly one third (28%; 11/39) would recommend low-dose aspirin (≤100 mg), 23% (9/39) would recommend >100 mg - 325mg, and 18% (7/39) would recommend 600 mg (**Table 3**; **Supplementary Table 4S**). Close to half of the respondents (47%, 26/55) would recommend beginning a patient with a LS diagnosis on aspirin at the commencement of the patient’s first screening colonoscopy (usually at age 25yrs) (**Table 3**; **Supplementary Table 5S**). Most participants (70%, 41/58) agree or strongly agree to recommend a low-dose of aspirin for patients with LS age 50-59y with cardiovascular disease risk of ≥ 10% over the next 10 years (**Table 3**).

**Table 3:** Chemoprevention clinical practices of providers with regards to their patients with LS.

Is chemoprevention used frequently in your patients with LS?		
	n=59	
	n	%
Yes	35	59.3
No	18	30.5
Uncertain, chemoprevention patient use, in any capacity, is outside the purview of my services	3	5.1
Other:	3	5.1
If yes, which dose per day?		
	n=39	
	n	%
≤100 mg	11	28.2
>100 mg - 325 mg	9	23.1
600 mg	7	1.8
Other (see **Supplementary Table 4S**)	12	30.8
Is chemoprevention discussed with your patients with LS?		
	n=59	
	n	%
Yes	40	67.8
Yes, I discuss it, but I am not the prescriber	15	25.4
No	3	5.1
Uncertain, chemoprevention is not within the purview of my services	1	1.7
Other	0	0.0
If aspirin has been advised for your LS patient, which of the following scenario applies you:		
	n=55	
	n	%
Begin aspirin from commencement of their colonoscopy screening (usually at age 25 years)	26	47.3
Begin aspirin under age 18 years	2	3.6
Begin aspirin at age 18-25y	6	10.9
Begin aspirin over age 25y	7	12.7
All ages would be considered	4	7.3
N/A	2	3.6
Uncertain	5	9.1
Other (see **Supplementary Table 5S**)	3	5.5
When your LS patient reaches 50-59 years of age and cardiovascular disease risk is ≥ 10% over the next 10 years, will you recommend low-dose of aspirin?		
	n=58	
	n	%
Strongly agree	23	39.7
Agree	18	31.0
Neutral	6	10.3
Disagree	5	8.6
Strongly disagree	1	1.7
Uncertain, in depth chemoprevention discussion is not within the purview of my services	5	8.6

### Factors influencing chemoprevention in LS (Case 4)

**Figure 6:**
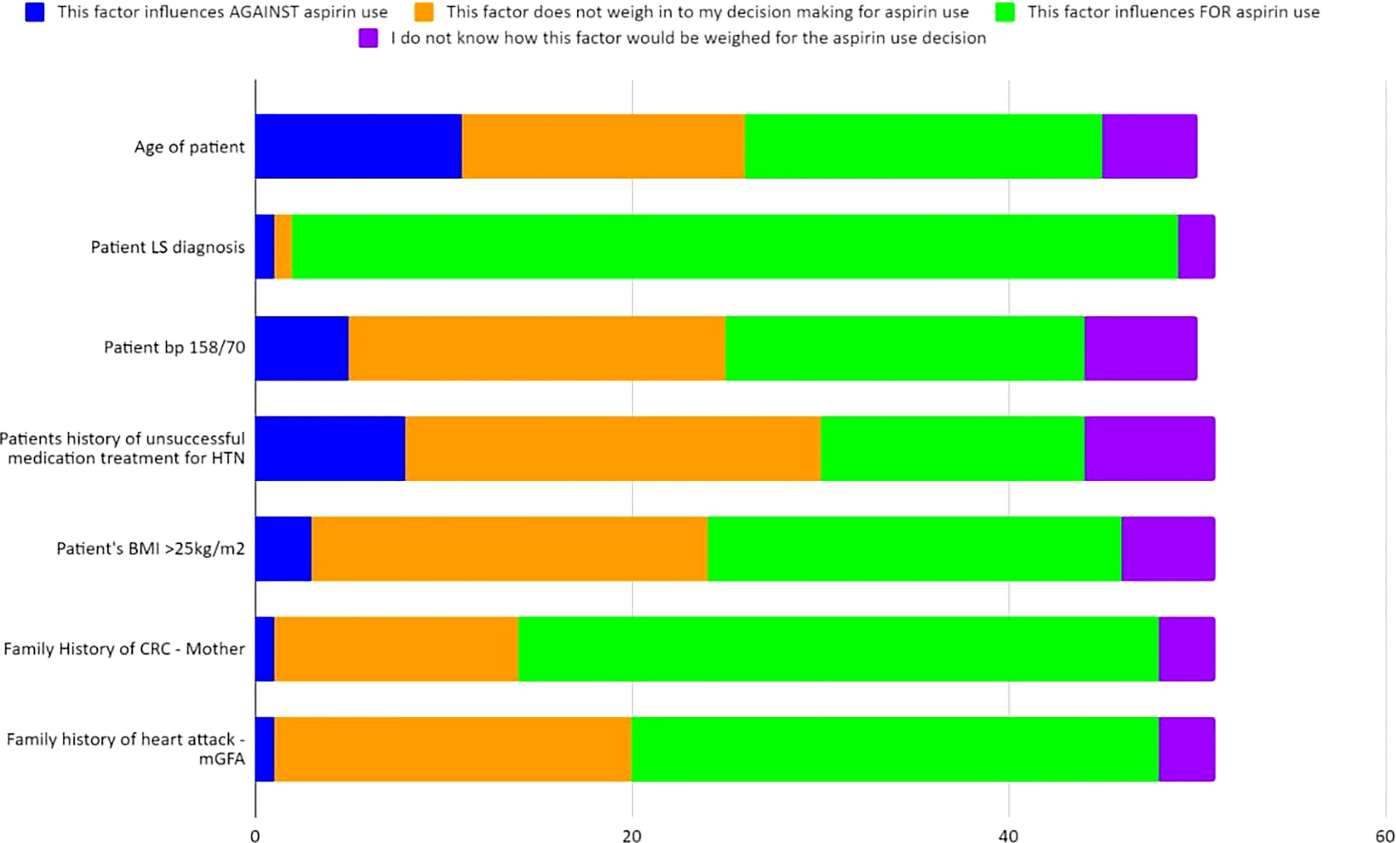
Case 4 - Responses of up to 50 participants from the four professional societies regarding how multiple factors weigh into their chemoprevention decision-making for a patient with LS such as that in Case 4 (further detailed in **Supplementary Table 6S**).

Case 4 described a 31-year-old female with an *MSH2* pathogenic variant, a personal health history of uncontrolled hypertension, and BMI >25kg/m^2^; she had a reported family history of a mother recently diagnosed with CRC and a paternal grandfather who died of a heart attack at age 52 (**Supplementary Survey**). Sixty percent (58/96) of the respondents answered at least one question for Case 4. Most respondents (62%, 36/58) would offer chemoprevention to the patient described in Case 4 with LS (**Table 4**; **Supplementary Table 6S**). Multiple factors were assessed for their influence on aspirin recommendation for LS patients (**Figure 6**; **Supplementary Table 6S**). Ninety-four percent of respondents (47/50) considered the patient’s diagnosis of LS to be an influencing factor for aspirin use. Sixty-eight percent (34/50) of respondents reported that family history of CRC (in mother) would influence *in favor of* aspirin use, though close to one-third (26%, 13/50) thought this *did not* influence their recommendation. Similarly, family history of heart attack in a second degree relative was largely seen as an influencing factor *for* aspirin use by 56% (28/50), while 38% (19/50) indicated this history would *not* influence their decision to offer aspirin. Respondents were also divided on how age, hypertension, and high BMI should influence the recommendation for aspirin use with each of these factors having a close to an even split as either being considered as a factor that influences *for* aspirin use or *not weighing* into the respondent’s decision making for aspirin use. There was notably no consensus regarding how the patient‘s age of 31 years would factor into the aspirin use decision with 38% *for* aspirin use, 30% *not weighing* it as a factor, 22% *against* aspirin use and 10% *did not know*. Fifty-one respondents answered additional questions regarding long-term use of 600 mg and 150 mg and its ability to reduce the risk for the patient described in Case 4. Eighty-two percent (42/51) agree or strongly agree that 600 mg aspirin will reduce this patient’s risk whereas only 41% (21/51) agree or strongly agree that 150 mg would reduce this patient’s risk (**Table 4**).

**Table 4:** Case 4 - Chemoprevention strategies for LS.

Case 4 Inquiries:		
Do you recommend the uptake of aspirin for this patient?		
	n=58	
	n	%
Yes	36	62.1
No	8	13.8
Uncertain, in depth chemoprevention discussion, **in any capacity**, is not within the purview of my practice	6	10.3
Other(See **Supplementary Table 6S**)	8	13.8
A long term-intake of daily 600 mg aspirin will reduce the risk of this patient?		
	n=51	
	n	%
Strongly agree	21	41.2
Agree	21	41.2
Neutral	1	2.0
Disagree	3	5.9
Strongly disagree	0	0.0
Uncertain	5	9.8
A long term-intake of daily 150 mg aspirin will reduce the risk of this patient?		
	n=51	
	n	%
Strongly agree	5	9.8
Agree	16	31.4
Neutral	7	13.7
Disagree	5	9.8
Strongly disagree	1	2.0
Uncertain, in depth chemoprevention discussion is not within the purview of my services	17	33.3

## Discussion

Hereditary CRC syndromes are being increasingly diagnosed and the use of chemoprevention as part of a comprehensive risk management strategy for affected individuals remains controversial. In this study that surveys providers with expertise in hereditary CRC syndromes from four international societies, we provide a global perspective on the patterns, preferences and potential implementation barriers regarding chemoprevention for FAP and LS patients in clinical practice. To our knowledge, this is the first study to ascertain the chemoprevention care approach for the two most common hereditary CRC syndromes.

Most of the respondents (66%) perceive that there is patient interest in chemoprevention and 88% reported this as the area of major clinical interest. Sixty-nine percent of the respondents offered chemoprevention to patients with FAP and/or LS as a part of their practice. This is in contrast with a previous study that described a limited awareness of LS and preventive effects of aspirin among general practitioners (10). Respondents in this study may have a higher endorsement and knowledge in chemoprevention practice than other studies based on the hereditary cancer expertise of the participating societies.

### FAP chemoprevention

Regarding rectal polyposis management, varying chemoprevention recommendations in national guidelines and consensus statements utilized by hereditary GI cancer providers (**Supplementary Table 1S**) highlights a lack of consensus for type and dosage of agents appropriate for chemoprevention, as well as when chemoprevention should be used. Given the history and risk of developing post-surgical desmoid tumors for the FAP patient in Case 1, it was not surprising that despite the rectal polyposis, only 36% (35/96) the responses included a surgical management option (**Supplementary Tables 2S-2.1S**). The most common non-surgical management selection was a combination of chemoprevention with endoscopic surveillance of the rectum. Low-dose NSAIDs and COX-2 inhibitors have been seen to have a dual benefit for reduction of polyp burden and have been reported in desmoid management, though data on low-dose aspirin is limited (15, 17, 20). Respondents selected sulindac and aspirin as the top considerations for chemoprevention agents for rectal polyposis in the adult FAP vignette; both agents have been reported in the literature (15).

Chemoprevention studies for FAP have also been performed in the pediatric setting. Specifically, Celecoxib - 400mg has been studied in children ages 10-14yrs which showed no adverse effects, a 44% reduction in polyps (21) as well as the CAPP1 study described above (16). Only 30% of our participants (who consider chemoprevention in their purview) would offer chemoprevention to the pediatric FAP patient in Case 2 and 35% respondents indicated they’d offer chemoprevention to a specific age group (**Table 2**). Due to possible unforeseen biases *via* case presentations, it is possible that some respondents may not have recommended chemoprevention to the pediatric patient presented in Case 2 but do offer chemoprevention to certain age groups of FAP patients that were not captured due to study design. With regards to our participants’ patients, there is no consensus age group where chemoprevention is offered, which may be due to the variable age of onset of polyposis in FAP.

### Lynch syndrome chemoprevention

As seen in **Table 1S** of the Supplementary, there is no consensus on CRC chemoprevention recommendations for LS. The BSG/ACPGBI/UKCGG, Cancer Council of Australia (CCA), EHTG/ESCP, NICE, and NCCN recommend that individuals with LS should be advised to have regular aspirin use for CRC risk reduction; notably NCCN and NICE both recommend to “consider” aspirin use (19, 22–25). Furthermore, EHTG/ESCP makes a recommendation regarding a minimum dosage of 75-100mg daily which should be increased for people with above-average body mass (25) and the CCA Clinical Guidelines has a recommendation regarding the age to start aspirin in LS (at time of first colonoscopy, typically at 25y) (24). Of note, the CCA also has recommendations regarding CRC chemoprevention (via aspirin) for both non-syndromic conditions and the general population in addition to a particular practice point regarding uncontrolled hypertension and aspirin (24). The USMSTF and ASCO (in agreement with ESMO guidelines) currently state to “consider” aspirin use for LS patients with an emphasis of limited data (26, 27). ACG and ESDO reference the data but do not make a current recommendation for aspirin for LS patients, though ESDO does recommend a critical discussion of the CAPP2 trial data (28, 29). Our study found that 59% of respondents frequently *use* chemoprevention for their LS patients and 93% *discuss* it with their patients (**Table 3**; **Figure 5**). Overall, our study population does not have a consensus on daily aspirin dosing (**Table 3**; **Supplemental Table 4S**). There was consistency regarding the safety barrier of age and cardiovascular risk and not prescribing past age 70, which is consistent with the CAPP2 study (30). Of great interest will be the results on the ongoing CAPP3 study which aims to assess whether lower doses of aspirin (100 mg, 300 mg) have similar efficacy to 600 mg of daily aspirin on CRC incidence in LS.

Case 4 allowed for analysis of the factors that may influence the decision-making process for chemoprevention for patients with LS, specifically those with a high BMI, cardiac risk factors (uncontrolled HTN; family history of heart attack) and other LS-related factors (FMH of CRC). As detailed in **Table 4**, 62% of the respondents would recommend aspirin for this patient. Further investigation of factors that influence decision-making highlighted several areas of uncertainty (**Figure 6**; **Supplementary Table 6S**). Respondents from the four international hereditary CRC groups largely agreed (94%; 47/50) that the patient’s diagnosis of LS influenced for aspirin use (**Figure 6**; **Supplementary Table 6S**). This was an expected response given the CAPP2 trial publication on 10-year data on aspirin benefit in the LS population and numerous guideline publications recommending aspirin as a form of chemoprevention for these patients (23, 28, 31). There was not, however, a consensus regarding whether other factors would influence chemoprevention use. Prior guidelines *for aspirin* use that address cardiovascular disease (CVD) prevention took the gender differences into consideration: it is typically intended for the prevention of coronary artery disease in males and the prevention of strokes in females (32). However, a recent guideline update in 2022 recommended the decision to initiate low-dose aspirin use for primary prevention of CVD in adults 40-59 yrs of age who have a 10% or greater 10-year CVD risk should be an individual one; additionally the guidelines recommended against the initiation of low-dose aspirin use for the primary prevention of CVD in adults ≥60 years (33).

The CAPP2 randomized trial studied rates of CRC in individuals with LS who took 600 mg of aspirin versus placebo. Obesity was found to be an independent risk factor for CRC and can be mitigated with aspirin, specifically highlighting that aspirin may be most beneficial in obese patients with LS (30). Interestingly, there was a close to even split with nearly 40% of respondents who felt that a BMI>25kg/m2 either *did not weigh* into the decision-making process of aspirin use or would influence *in favor* of aspirin use; this was a similar split for impact of hypertension on chemoprevention decision-making. This was a surprising finding given that the study respondents are from hereditary cancer societies and are likely aware of the CAPP2 trial data.

Given the substantial implications of aspirin use in cardiovascular diseases in addition to its beneficial effects on adenoma rate and CRC, along with its potential risks (such as gastrointestinal hemorrhage), inclusion of cardiologists and gastroenterologists in the decision-making process may be prudent, particularly when deciding whether the higher dosage (300 mg - 600 mg) aspirin should be considered (34).

A much higher percentage of our participants agreed or strongly agreed that the 600 mg aspirin would be recommended to reduce the risk in the LS patient described in Case 4, however, there was notable discordance regarding aspirin dose for the LS patient in Case 3 among our respondents. It is striking that there is an overall lack of uniform consensus across the cases with regards to chemoprevention dose, type, and in the case of aspirin, factors that may influence recommendations. This study is the first of its kind to review international practices on chemoprevention while highlighting the uncertainty and need for more quality studies and will require guideline forming bodies to continue to address this issue.

### Barriers to chemoprevention

The main barrier reported by study participants was the possible harm of aspirin use among older (>70 years) adults. This is in line with the unknown knowledge about the long-term durability and safety of many agents ^16^. Currently, there are no chemoprevention agents approved by the Food and Drug Administration (FDA) for hereditary CRC syndromes.

Our findings highlight the need for long-term durability, safety studies, and a clear understanding of how and which cardiovascular factors may play a role in the decision-making process for aspirin use when evaluating for chemoprevention. Future studies may allow for bottleneck routing of participants based on cumulative selections.

### Limitations

There are several limitations to our study. Although there was positive feedback from the participating international societies, half of the respondents belonged to CGA-IGC which could limit the generalizability of the results, as CGA-IGC members are mainly US based. In addition, the current approach for chemoprevention in LS and FAP patients were based on clinical cases, and therefore there may be other important aspects of the chemoprevention issue(s) that were not assessed by the cases used. Also, while the participants are from hereditary colorectal cancer societies, it is unknown what portion, if any, regularly manage pediatric FAP cases within their practice. The practice limits of participants including but not limited to age limits of patients may also have been a limitation for this study. One further limitation of this study is that respondents who selected “Strongly Agree” *and/or* “Uncertain” to *all* of the barrier questions were to be rerouted and bypass the vignettes. However, upon review, respondents‘ answers did not reflect their comprehensive barrier selections and thus some were routed to the end of the survey when they had met criteria to complete vignettes which resulted in reduced responses for Case vignette data.

## Conclusion

Chemoprevention is widely offered for patients with LS and FAP in clinical practice based on this international survey. However, substantial heterogeneity remains with regard to patient selection and optimal agents and dosage for chemoprevention, calling for further investigations.

## Data availability statement

The original contributions presented in the study are included in the article/**Supplementary Material**. Further inquiries can be directed to the corresponding author.

## Ethics statement

The study involving human participants were reviewed, granted an exemption and approved by the Regional Committee for Medical and Health Research Ethics, Section D, South East Norway. The research project was assessed in accordance with the Norwegian Research Ethics Act 2006 and Act on Medical and Health Research 2008. The patients/participants provided their electronic written informed consent to participate in this study.

## Author contributions

KM, RH, LR-F, BK, and MD-V designed the study. KM and MD-V performed the analysis and wrote the manuscript. All authors have read and agreed to the final version of the manuscript.
