# Supplementary material for: Current chemoprevention approaches in Lynch syndrome and Familial adenomatous polyposis: a global clinical practice survey
Source: Front Oncol. 2023 May 24;13:1141810. doi: 10.3389/fonc.2023.1141810 (PMC10247284; doi:10.3389/fonc.2023.1141810)
Supplement: Supplementary file 1 [file DataSheet_1.pdf]

Table 1S: Current CRC Chemoprevention Recommendations for Patients with LS and FAP by National Guidelines/Professional Organizations

Organization	Condition	Recommendation/Actively Consider	Consideration/Suggest or Mentioned in Prevention discussion Summary (if no recommendation provided)
**ACG** [1]	LS	• Currently the evidence is not sufficiently robust or mature to make a recommendation for its standard use (conditional recommendation, moderate quality of evidence)	
	FAP	Not provided	• Sulindac - considerable regression and prevention of colonic and rectal adenomas has been demonstrated; not considered a substitute for colectomy but has shown utility in rectal surveillance by substantially decreasing the number of adenomas needing removal at periodic examination.<br>• Celecoxib - was approved for use in the U.S for several years for FAP due to more modest effect in the colon and rectum and some effect for duodenal adenoma regression, but due to cardiovascular side effects of long -term COX-2 inhibitors has lead to removal of this indication.<br>• Nonsteroidal anti-inflammatory drugs and other agents: TBD
**ASCO** [2]	LS	Not provided.	• The ASCO endorsement panel agrees with the ESMO guidelines that the use of aspirin may be considered for cancer prevention; however, because existing data on the effectiveness of aspirin for cancer prevention in LS are derived from a single clinical trial, there are insufficient data to make strong recommendations in favor of or against the use of aspirin for chemoprevention in LS.
	FAP	Not provided	• Primary chemoprevention has not been demonstrated in randomized controlled trials to delay the appearance of clinically significant polyposis in FAP.<br>• Nonsteroidal anti-inflammatory drugs has been shown to reduce the number and extent of colorectal adenomas and, less reliably, duodenal adenomas<br>• Sulindac and Celecoxib can be considered as adjuvant treatments when adenoma recurrence is detected in individuals who have undergone colectomy. Because

			cardiovascular adverse effects have been reported in patients receiving nonsteroidal anti-inflammatory drugs (including COX-2 inhibitors), caution is warranted
**BSG/ACPGBI/ UKCGG** [3]	LS	• Recommend that individuals with LS should be advised that regular use of daily aspirin reduces CRC risk. (GRADE of evidence: moderate; Strength of recommendation: strong)	
			• Suggest that people with LS should be offered research opportunities to take aspirin daily at different dosages. If they decline research participation they may be advised on their choices regarding dose of aspirin, risks and benefits of long-term aspirin use and ensure their medical practitioner is aware of their intake. (GRADE of evidence: low; Strength of recommendation: weak)
	FAP	• There is insufficient evidence of the benefit of chemoprophylaxis in polyposis syndromes. (GRADE of evidence: moderate; Strength of recommendation: strong)	
	Individuals at increased familial risk of CRC		• Suggest that these individuals should be strongly encouraged not to smoke, to maintain a normal body mass index (BMI), to moderate their consumption of red and processed meat, and to exercise regularly. (GRADE of evidence: low; Strength of recommendation: weak)
**Cancer Council Australia** [4]	All people	• Aged 50–70 years who are at average risk of CRC, aspirin should be actively considered to prevent CRC. A low dose (100–300 mg/d) is recommended for at least 2.5 years, commencing at age 50 - 70 years. The benefit may extend to older ages with longer duration of use. Benefit for cancer prevention (though shorter for cardiovascular risk) is evident only 10 years after initiation so a life expectancy of at least 10 years should be taken into consideration in the advice to use aspirin. The choice to take aspirin should be personalised based on age, sex and potential reduction in cardiovascular events, cerebrovascular events and thrombotic stroke. The individual should take into account the potential risks of taking aspirin. Aspirin should be avoided in patients with current dyspepsia, any history of peptic ulcer, aspirin allergy, bleeding diathesis, an increased risk of gastrointestinal haemorrhage (such as associated with use of oral anticoagulants or antiplatelet agents), or renal impairment.<br><br>The benefit in colorectal cancer risk reduction in women >65 is less clear cut. However, based on limited data available, older women with cardiovascular risk factors may derive a greater overall benefit than harm.<br><br>• Practice point: Aspirin should be avoided in patients with uncontrolled hypertension.	• Practice point: Breath testing for *Helicobacter pylori* (and treatment for those who test positive) can also be considered, as gastrointestinal toxicity from aspirin is enhanced in the presence of *Helicobacter pylori*.

	LS	• People who are at high risk of colorectal cancer due to Lynch Syndrome carrier status should be advised to begin aspirin from the commencement of their colonoscopy screening (usually at age 25 years).	
	Non-syndromic familial cancer	• Non-syndromic familial cancer patients should be actively considered for aspirin, bearing in mind the possibility of adverse events.<br>• 600 mg/day has been shown to be effective, but lower dose (100 mg/day) may be as effective and is recommended based on the data available at the time of the systematic review.	
	FAP	• Practice point: Where surgery is inappropriate for people with familial adenomatous polyposis, an NSAID (e.g. sulindac) is recommended).<br>• Practice point: Without RCT evidence, statins cannot be recommended for chemoprevention at this time.<br>• Practice point: Without RCT evidence, metformin cannot be recommended for chemoprevention.<br>• Practice point: Bisphosphonates cannot be recommended for chemoprevention.	
**ESDO** [5]	LS	• Critical discussion is warranted of the CAPP2 trial which showed a reduction of the LS-associated cancers with a 600mg/d of aspirin for at least 2 years; it is unknown whether high-dose aspirin (600 mg/d) is necessary to prevent further LS-associated cancers as the inhibition of cyclooxygenase-2 and subsequently decreased prostaglandin E2 synthesis- often the presumed mechanism of the prevention impact of aspirin on different malignancies- requires less than 100 mg daily. Recommendation also highlights dose non-inferiority studies comparing 100 mg, 300mg, and 600 mg of daily aspirin for prevention of LS associated malignancies in LS carriers (i.e. CAPP-3 and AAS-Lynch) that are underway to address this question.<br>• Patients should be advised to stop smoking, reduce overweight and increase physical activity. Nutritional counselling can be offered.	
	FAP	Not provided	

**EHTG/ ESCP** [6]	LS	• Path_MMR carriers should be advised that daily acetylsalicylic acid intake will reduce colorectal cancer risk.<br><br>• The recommended acetylsalicylic acid dose should be a minimum of 75–100 mg daily. This dose should be increased for people with above-average body mass.	
	FAP	Not provided	
**NCCN** [7]	LS	• Recommends that all individuals with LS who have a risk for future CRC (i.e., excluding those with prior total proctocolectomy) consider using daily aspirin to reduce their future risk of CRC.<br><br>• The decision to use aspirin for reduction of CRC risk in LS and the dose chosen should be made on an individual basis, including discussion of individual risks, benefits, adverse effects, and childbearing plans.<br><br>In determining whether an individual with LS should take aspirin and in deciding on the appropriate dosing, the panel recommends that providers carefully review patient specific factors that may increase the risk of aspirin therapy—including but not limited to increased age, prior allergy, concurrent use of antiplatelets/ anticoagulants, and untreated H. pylori or unconfirmed H. pylori eradication—as well as patient-specific factors that indicate a comparably low future cumulative risk of CRC (ie, increased age, PMS2-associated LS, history of prior colectomy) and who may thus be less likely to experience significant benefit.	
	FAP		• Chemoprevention may be considered to facilitate management of the remaining rectum or pouch post surgery in select patients with progressive polyp burden (e.g., based on size, number, and pathology).<br><br>• There are no FDA-approved medications for this indication at present.<br><br>• While there are data to suggest that sulindac is the most potent polyp regression medication, it is not known if the decrease in polyp burden decreases cancer risk.<br><br>• Patients interested in chemoprevention may consider referral to an expert center and enrollment in a clinical trial<br>.

**NICE** [8]	LS	• For Reduction in risk of colorectal cancer in people with Lynch syndrome, per the NG151 guideline section 1.1.1, they recommend to consider daily aspirin, to be taken for more than 2 years, to reduce the risk of colorectal cancer in people with Lynch syndrome. NICE has also created a patient decision aid to support discussions about taking aspirin.	
**USMSTF** [9]	LS		• Growing but not conclusive evidence exists that use of aspirin is beneficial in preventing cancer in LS patients.<br>• Treatment of an individual patient with aspirin is a consideration after discussion of patient specific risks, benefits, and uncertainties of treatment is conducted
	FAP	Not provided	

The above table depicts guidelines regarding chemoprevention by various professional organizations and societies, specifically:

The American College of Gastroenterology (ACG), American Society of Clinical Oncology (ASCO), the British Society of Gastroenterology/Association of Coloproctology of Great Britain and Ireland (BSG/ACPGBI/UKCGG), the Cancer Council of Australia (CCA), the European Society of Digestive Oncology (ESDO), the European Hereditary Tumour Group (EHTG) and the European Society of Coloproctology (ESCP), the National Comprehensive Cancer Network (NCCN), the National Institute for Health and Care Excellence (NICE), and the US Multi-Society Task force (USMSTF)

Survey

CGA Clinical Practice Survey 2021: Chemoprevention for Lynch syndrome and Polyposis

We would like to find out whether your service recommends chemoprevention for Lynch Syndrome (LS) and Familial Adenomatous Polyposis (FAP) patients. We are looking for the participation and response of healthcare providers who manage patients with LS and FAP.
The survey questions include the description of some barriers for chemoprevention practice and whether chemoprevention is offered/considered for LS and FAP patients, when chemoprevention prescribing, medications prescribed, age groups considered for chemoprevention and some clinical-based cases. This will help us understand the current chemoprevention practice for these patients among the CGA members.

We would be most grateful if you could answer these questions, it should not take any longer than 10 minutes to complete.

Are you a member of CGA-IGC?

- ☐ Yes
- ☐ No

Have you previously taken this survey? (if you are a CGA member, you may have already taken the survey)

- ☐ Yes (no proceed the survey)
- ☐ No (proceed to Question #1)
- ☐ Unsure (proceed to Question#1)

A. *Practice Characteristics*

1. What is your primary work setting?
   a. Academic Medical Center with patient care practice
   b. Academic Medical Center without patient care practice
   c. Community Hospital based practice
   d. Private patient care practice
   e. Other (fill in)

2. What is your primary specialty/role?
   a. Genetic Counselor in Cancer Genetics
   b. Genetic Counselor in Hereditary GI only
   c. Gastroenterologist
   d. GI Oncologist
   e. Medical oncologist
   f. Colorectal surgeon
   g. Thoracic surgeon
   h. Surgical oncologist
   i. General surgeon
   j. Gynecologic oncologist
   k. Gynecologist
   l. Primary care provider

m. Researcher
n. Nurse
o. Medical Geneticist
p. Employee at a genetic testing laboratory
q. Other (fill in)

3. Primary location of where the patients you treat reside

[Drop-down menu of all countries]
[If USA chosen, drop down menu for each state]

4. How long has your practice/group been providing hereditary GI cancer risk assessment?
a. Less than 5 years
b. 5-10 years
c. 10 years or more

5. Approximately how many LS and/or FAP patients do you attend to per week?
a. Less than 5
b. 5-10
c. More than 10
d. N/A

6. Does your service offer chemoprevention for LS and/or FAP patients as a part of your practice?
a. Yes
b. No (Go to Question 9)
c. Other (specify)

7. Which year did your service start prescribing chemoprevention?
Include option of "Don't know/Uncertain"
[Drop down menu with years from 1980-present]

B. Current barriers for chemoprevention practice in LS and FAP syndromes

8. My patients are not interested in chemoprevention
a. Strongly agree
b. Agree
c. Neutral
d. Disagree
e. Strongly disagree
f. Uncertain, chemoprevention discussions are not within the purview of my services

9. There are no satisfactory referral resources available for chemoprevention in LS and FAP
a. Strongly agree
b. Agree
c. Neutral
d. Disagree

e. Strongly disagree
f. Uncertain, chemoprevention discussions are not within the purview of my services

10. There is insufficient reimbursement for chemoprevention activities in my clinical practice
    a. Strongly agree
    b. Agree
    c. Neutral
    d. Disagree
    e. Strongly disagree
    f. Uncertain, in depth chemoprevention discussions are not within the purview of my services

11. I need more information to be comfortable providing chemoprevention recommendations
    a. Strongly agree
    b. Agree
    c. Neutral
    d. Disagree
    e. Strongly disagree
    f. Uncertain, chemoprevention discussions are not within the purview of my services

12. Chemoprevention is not an area of major clinical interest to me
    a. Strongly agree
    b. Agree
    c. Neutral
    d. Disagree
    e. Strongly disagree
    f. N/A, chemoprevention is not within the purview of my services

13. Chemoprevention is too time consuming to be economically included in my clinical practice
    a. Strongly agree
    b. Agree
    c. Neutral
    d. Disagree
    e. Strongly disagree
    f. Uncertain, chemoprevention (regardless of depth) is not within the purview of my services

14. We don’t know the effectiveness of aspirin chemoprevention for the endpoints of reduction in incidence of CRC or mortality of CRC.
    a. Strongly agree
    b. Agree
    c. Neutral
    d. Disagree
    e. Strongly disagree
    f. Uncertain, in-depth chemoprevention knowledge is not within the purview of my services

15. We don't know the harms of aspirin chemoprevention for the endpoints of reduction in incidence of CRC or mortality of CRC.
   a. Strongly agree
   b. Agree
   c. Neutral
   d. Disagree
   e. Strongly disagree
   f. Uncertain, in-depth chemoprevention knowledge is not within the purview of my services

16. There is a lack of benefit among older (>70y) adults to use aspirin.
   a. Strongly agree
   b. Agree
   c. Neutral
   d. Disagree
   e. Strongly disagree
   f. Uncertain, in-depth chemoprevention knowledge is not within the purview of my services

17. There is possible harm among older (70y) adults to use aspirin.
   a. Strongly agree
   b. Agree
   c. Neutral
   d. Disagree
   e. Strongly disagree
   f. Uncertain, in-depth chemoprevention knowledge is not within the purview of my services

C.1. Current chemoprevention recommendations for FAP patients

If you have selected ONLY "Strongly agree" AND/OR "Uncertain" to <u>ALL</u> of the barrier questions 8-17 in section B.1, it suggests that either your service/center does not prescribe or support the use of chemoprevention AND/OR chemoprevention knowledge is not within the purview of your services. Then, section C.1 and C.2 will not apply for you. Please proceed to section D.

<u>CASE 1</u>: A 33 yo male with FAP comes to your office to establish care. He underwent subtotal colectomy with an ileorectal anastomosis at age 21, and he has a 2 cm intra-abdominal desmoid that was diagnosed 5 years ago, which has remained stable in size on surveillance. He has 10cm of rectum remaining, with ~100 polyps ranging in size from 2-10 mm. He has Spigelman stage II duodenal polyposis, and no other medical problems.

18. Based on the provided history, what would you recommend for management of your patient's rectal polyp burden? (SELECT ALL THAT APPLY)

   a. Completion colectomy with ileal pouch anal anastomosis
   b. Completion colectomy with end ileostomy
   c. Endoscopic surveillance of the rectum

d. Chemoprevention
e. Other (specify)
f. Uncertain

19. Does your service provide/offer chemoprevention to this patient?
    a. Yes (go to Question 19)
    b. No, (Go to Case 2)
    c. N/A (specify)
    d. Uncertain, chemoprevention discussion, <u>in any capacity</u>, is outside the purview of my services (Go to Case 2)

20. If yes, what type? What total daily dosage?

		Sulindac
		150 mg
		200 mg
		300 mg
		600 mg
		Other (specify)
		Don't know/ uncertain
		Combination (difluoromethylornithine (DFMO) plus sulindac), dosage unknown
		Aspirin
		Tiracoxib
		Celecoxib
		Rofecoxib

	Erlotinib
	Natural compounds
	Other (specify)

CASE 2: A family is referred for genetic counseling due to a strong history of FAP and had a confirmed *APC* pathogenic variant. The family history includes two sisters who had colectomies at 22 and 23 who are now in their 30's. Their mother was confirmed to have an *APC* pathogenic variant and had a manageable case of polyposis up until age 60 when her GI decided to pursue colectomy. Your clinic is following the children of each of the sisters. One of the children is now 15 years old and began undergoing colonoscopy at age 8y due to blood in the stool. He had 5 adenomatous polyps at his first colonoscopy. He continued to have colonoscopies. Now fast forward to age 15, since the time of his first colonoscopy, his polyp burden has consistently increased. He has been having colonoscopies every year but skipped last year. Review of his endoscopy note showed carpeting of polyps throughout his transverse colon that extends to part of his cecum and into his sigmoid colon. His rectum has 14 polyps. Two large polyps, one of which was 1.2 cm but all were tubular adenomas based on the sampling that was provided to pathology.

21. Does your service provide/offer chemoprevention to this patient?
   a. Yes (go to Questions 21 and 22)
   b. No (Go to Case 3)
   c. N/A (specify)
   d. Uncertain, chemoprevention discussion, in any capacity, is outside the purview of my services (Go to Case 3)

22. Is chemoprevention offered to a specific age group for your FAP patients?
   a. Yes (go to Question 22)
   b. No (go to C.2)
   c. Uncertain (go to Question 22)
   d. N/A (specify)

23. Which age group (years)?
   a. <18y
   b. 18y-25y
   c. 26y-35y
   d. 36y-45y
   e. >45
   f. Other (specify)

C.2. Current chemoprevention recommendations for LS patients

CASE 3: A 45 y.o. female with a personal history of metachronous colorectal cancers (dx at 27 and 32) is seen today for updated recommendations regarding her *MSH2* pathogenic variant which has since been confirmed to be maternally inherited. Although the patient has a history of colon cancers, she has much of her colon intact and is quite vigilant in LS surveillance. She has no other significant health conditions in her or her family.

24. Based on the provided history, what would you recommend for your patient? (SELECT ALL THAT APPLY)
   a. Colectomy
   b. Total proctocolectomy
   c. No surgery
   d. Endoscopic surveillance
   e. Chemoprevention
   f. Other (specify)
   e. Uncertain

25. Is chemoprevention used frequently in your patients with LS?
   a. Yes
   b. No, go to Question 26
   c. Other (specify)
   d. Uncertain, chemoprevention patient use, in any capacity, is outside the purview of my services (Go to Question 26)

26. If yes, which dose per day:
   a. 100 mg
   b. 300 mg
   c. 600 mg
   d. Other (specify)

27. Is chemoprevention discussed with your patients with LS?
   a. Yes
   b. Yes, I discuss it, but I am not the prescriber
   c. No (go to Question 28)
   d. Uncertain, chemoprevention is not within the purview of my services (go to Question 28)

28. If aspirin has been advised for your LS patient, which of the following scenario applies you:
   a. Begin aspirin from commencement of their colonoscopy screening (usually at age 25 years)
   b. Begin aspirin under age 18 years
   c. Begin aspirin at age 18-25y
   d. Begin aspirin over age 25y
   e. All ages would be considered
   f. Other (specify)
   g. N/A
   h. Uncertain

29. When your LS patient will reach 50-59 years of age and may have a cardiovascular disease risk of ≥ 10% over the next 10 years, will you recommend a low dose of aspirin?
   a. Strongly agree
   b. Agree
   c. Neutral
   d. Disagree
   e. Strongly disagree
   f. Uncertain, in-depth chemoprevention discussion is not within the purview of my services

CASE 4: A 31 y.o. female with LS due to a pathogenic *MSH2* variant is seen for evaluation. She denies family history of cancer outside of her mother who was recently diagnosed with colon cancer but through further workup was found to have endometrial cancer. Patient's maternal grandfather died at 52 from a heart attack. Patient's blood pressure today is 158/92. You note this is on the high end (as it is above 130) and inquire about history of hypertension. She states that her primary care physician has had her on medication regarding the blood pressure for a trial period before, but it has not helped. She has a BMI above 25kg/m2.

30. Do you recommend the uptake of aspirin for this patient?
   a. Yes
   b. No (go to Question 30)
   c. Other (specify)
   d. Uncertain, in-depth chemoprevention discussion, in any capacity, is not within the purview of my practice (go to Part D)

31. How do you weigh each of the factors in your decision making?

	This factor influences AGAINST aspirin use	This factor does not weigh in to my decision making for aspirin use	This factor influences FOR aspirin use	I do not know how this factor would be weighed for the aspirin use decision
Age of patient				
Patient LS diagnosis				
Patient bp 158/70				

Patient's history of unsuccessful medication treatment for HTN				
Patient's BMI >25kg/m2				
Family History of colon cancer - mother				
Family History of heart attack - mGFA				
OTHER FACTOR (write in)				

32. A long term-intake of daily 600 mg aspirin will reduce the risk of this patient?
    a. Strongly agree
    b. Agree
    c. Neutral
    d. Disagree
    e. Strongly disagree
    f. Uncertain

33. A long term-intake of daily 150 mg aspirin will reduce the risk of this patient?
    a. Strongly agree
    b. Agree
    c. Neutral
    d. Disagree
    e. Strongly disagree
    f. Uncertain

D. Final Remarks

In some cases, we would like to clarify survey responses. If you agree to be contacted, please provide your contact details below.

Name:
Preferred method of contact:
Email:
Telephone:

Other contact details:

Future Correspondence

I wish to be informed of the study findings. (Please provide contact details)
I do not wish to be contacted for this specific study again.

Table 2S: Current chemoprevention practice for FAP patients (Supplementary to Figure 2)

Case 1 Inquiries:

Based on the provided history, what would you recommend for management of your patient's rectal polyp burden? SELECT ALL THAT APPLY.			N=63	
			n	%
Completion Colectomy w/ Ileal pouch anal anastomosis (IPAA) (ONLY)			19	30.2
Completion colectomy with end ileostomy (ONLY)			1	1.6
Endoscopic surveillance of the rectum (ONLY)			6	9.5
Chemoprevention (ONLY)			2	3.2
Uncertain (ONLY)			7	11.1
**Combinations:**				
Completion Colectomy w/ IPAA	Completion colectomy with end ileostomy		1	1.6
Completion Colectomy w/ IPAA	Endoscopic surveillance of the rectum		1	1.6
Completion Colectomy w/ IPAA	Chemoprevention		2	3.2
Completion Colectomy with end ileostomy	Endoscopic surveillance of the rectum		1	1.6
Completion Colectomy with end ileostomy	Chemoprevention		1	1.6
Endoscopic Surveillance of Rectum	Chemoprevention		16	25.4
Completion Colectomy w/ IPAA	Completion Colectomy with end ileostomy	Endoscopic surveillance of the rectum	1	1.6
Completion Colectomy w/ IPAA	Completion Colectomy with end ileostomy	Chemoprevention	1	1.6
Completion Colectomy w/ IPAA	Endoscopic surveillance of the rectum	Chemoprevention	3	4.8
Completion colectomy with end ileostomy	Endoscopic surveillance of the rectum	Chemoprevention	1	1.6

Table 2.1S: Case 1 Management modalities (continued from Table 2S) (Supplementary to Figure 2)

Other (specify)	Additional responses with "Other" selection (which is reflected in Table 2S)	N=7*
• Other this is not my expertise, I leave that to the gastroenterologist to decide	• Uncertain	1
• I do not see patients with this problem	• Uncertain	1
• I would review the case with colorectal surgeons	• Uncertain	1
• Initial attempts at endoscopic therapy of rectum, not just surveillance and then surgery if that was unsuccessful	• Endoscopic surveillance of the rectum	1
• Need to know optimal dose	• Endoscopic surveillance of the rectum<br>• Chemoprevention	1
• Would see how he responded before taking out the rectum, as long as not cancer or HGD	• Endoscopic surveillance of the rectum<br>• Chemoprevention	1
• Would want to see longitudinally the polyp evolution/changes before I truly recommended operation, but would still discuss the operative options with the patient to be complete (so would lean to endoscopic treatment/surveillance and chemoprevention first)	• Completion colectomy with IPAA<br>• Endoscopic surveillance of the rectum<br>• Chemoprevention	1

* 7/63 respondents described in Table 2S also selected “Other (specify)” in addition the modalities to manage the rectal polyposis for Case 1. Their responses are in this table along with the other selections that they chose (which are also depicted in Table 2S and Figure 2).

Table 2.2S: Chemoprevention specifics for Case 1 (Supplementary for Figure 3)

If chemoprevention would be offered, what type? What total daily dosage?			
			N=35
Types of chemopreventions to choose from below, if selected only ONE option, reflected in this section:			N=23
Sulindac - 150 mg			3
Sulindac - 200 mg			4
Sulindac - 300 mg			4
Sulindac - 600 mg			1
Combination (difluoromethylornithine (DFMO) plus sulindac), dosage unknown			1
Aspirin			6
Tiracoxib			0
Celecoxib			1
Rofecoxib			0
Erlotinib			0
Natural compounds			0
Other (specify)			
	Not applicable		*2*
	Clinical Trial		*1*
**If selected multiple selections, see below: * (indicates that respondent indicated that selections were indicative of chemoprevention to be considered but not necessarily to be used simultaneously)**			N=12
Sulindac-150mg	Sulindac-300mg		1
Sulindac-150mg	Aspirin		1
Sulindac-150mg	Celecoxib		1
Sulindac-150mg	Natural Compoundnd (EPA)		1*
Sulindac-150mg	Other (clinical trial)		1
Sulindac-300mg	Aspirin		1
Sulindac-300mg	Celecoxib		2
Sulindac-150mg	Sulindac-200mg	Combination (difluoromethylornithine (DFMO) plus sulindac), dosage unknown	1
Sulindac-150mg	Combination (difluoromethylornithine (DFMO) plus sulindac), dosage unknown	Erlotinib	1
Sulindac-300mg	Aspirin	Celecoxib	1*
Sulindac-300mg	Celecoxib	Other (Clinical trial)	1

Table 3S: Case 3 - Current chemoprevention practices for LS patients (Supplemental to Figure 4)

Case 3 Inquiries

Based on the provided history, what would you recommend for management of your patient's rectal polyp burden? SELECT ALL THAT APPLY.

			N=59	
			n	%
Completion Colectomy w/ (IPAA) (ONLY)			2	3.4
Completion colectomy with end ileostomy (ONLY)			1	1.7
No Surgery (ONLY)			1	1.7
Endoscopic surveillance (ONLY)			6	10.2
Chemoprevention (ONLY)			2	3.4
Uncertain (ONLY)			4	6. 8
**Combinations:**				
Completion Colectomy w/ IPAA	Completion colectomy with end ileostomy		0	0.0
Completion Colectomy w/ IPAA	Endoscopic Surveillance		1	1.7
Completion Colectomy w/ IPAA	Chemoprevention		0	0.0
Completion Colectomy w/ end ileostomy	Endoscopic Surveillance		0	0.0
Completion Colectomy w/ end ileostomy	Chemoprevention		0	0.0
Endoscopic Surveillance	Chemoprevention		33	55.9
No Surgery	Endoscopic Surveillance		1	1.7
Completion Colectomy w/ IPAA	Completion Colectomy with end ileostomy	Endoscopic Surveillance	0	0.0
Completion Colectomy w/ IPAA	Completion Colectomy with end ileostomy	Chemoprevention	0	0.0
Completion Colectomy w/ IPAA	Endoscopic Surveillance	Chemoprevention	2	3.4
Completion colectomy w/ end ileostomy	Endoscopic Surveillance	Chemoprevention	1	1.7
No Surgery	Endoscopic Surveillance	Chemoprevention	5	8.5

Table 4S: Follow up to Case 3 - Aspirin dose recommendations for LS patient(s) of participant(s)
(Supplementary to Table 3)

If yes, which dose per day?			
		N=39	
		n	%
100 mg		10	25.6
300 mg		5	12.8
600 mg		7	1.8
Other (specify)		17	43.6
	• Recommend 600 based on CAPP2, but 325 then 81 if 600 not tolerated<br>• I base the dose on the individual's gene-/age-/sex-specific future risk of colorectal cancer, including the amount of large bowel remaining (as well as risk factors for bleeding, such as age, gastritis, concurrent anticoagulants).<br>• Start with 600 mg, and decrease incrementally if not tolerated<br>• 325 mg and if well tolerated 650<br>• 325 mg/day or highest dose tolerated<br>• Start 81 mg, double to 162 then usually around 300-325 mg. Some patients choose to be closer to 600 mg<br>• Dosage depends on BMI 75mg or 150 if >BMI 30<br>• 650 mg (It is difficult to make 600 mg in the United States.)<br>• If you give it, you should use 600 mg as in the study; this is not available in NL<br>• Aspirin 325 mg daily<br>• 200 mg<br>• 162 mg<br>• 81 or 162 mg<br>• Aspirin 81 mg daily<br>• 81 mg<br>• Await capp3 re dose<br>• Unsure of this; discussion is had; dosage not discussed by me		

Table 5S: Case 3 – Chemoprevention strategies for LS, further detailed (Supplementary to Table 3)

If aspirin has been advised for your LS patient, which of the following scenario applies you:		
	N=55	
	n	%
Begin aspirin from commencement of their colonoscopy screening (usually at age 25 years)	26	47.3
Begin aspirin under age 18 years	2	3.6
Begin aspirin at age 18-25y	6	10.9
Begin aspirin over age 25y	7	12.7
All ages would be considered	4	7.3
N/A	2	3.6
Uncertain	5	9.1
Other (specify)<br>• I'm not the prescriber. I have a general convo about this with all LS pts at time of dx (since it may be the only time it gets brought up). This prompts discussion with GI specialist who prescribes when appropriate.<br>• I begin aspirin as soon as I meet someone (unless they have contraindications and/or are actively pursuing pregnancy). Usually, early adulthood<br>• Begin aspirin from commencement of their colonoscopy screening (usually age 18-25y)	3	5.5

Table 6S: Chemoprevention practice recommendations for LS for Case 4
(Supplementary to Table 4 and Figure 6)
Case 4 inquiries:

Do you recommend the uptake of aspirin for this patient?	N=58	
	n	%
Yes	36	62.1
No	8	13.8
Uncertain, in-depth chemoprevention discussion, **in any capacity**, is not within the purview of my practice	6	10.3
Other (Specify)	8	13.8

- Given the younger cardiac FMH and the prominent uncontrolled HTN in the pt, I'd actually want to make sure cardio eval was completed. If no additional recommendations, aspirin could be revisited due to the LS; of note, though low dose aspirin can lower HTN, typically do not start til older
- Needs BP controlled first
- Would want hypertension controlled first
- Would discuss at MDT before making decisions and have discussion with patient about risk factors
- Depends on comorbidities
- Vascular disease is not my specialty, dosing for vascular risk is different from aspirin dosing in chemoprevention in LS
- Uncertain, in-depth chemoprevention discussion, **in any capacity**, is not within the purview of my practice
- Await capp3

How do you weigh each of the factors in your decision making?

	This factor influences AGAINST aspirin use	This factor does not weigh in to my decision making for aspirin use	This factor influences FOR aspirin use	I do not know how this factor would be weighed for the aspirin use decision
Age of patient (N=49)	11 (22%)	15 (30%)	19 (38%)	5 (10%)
Patient LS diagnosis (N=50)	1 (2%)	1 (2%)	47 (94%)	2 (4%)
Patient bp 158/70 (N=49)	5 (10%)	20 (40%)	19 (38%)	6 (12%)
Patients history of unsuccessful medication treatment for HTN (N=50)	8 (16%)	22 (44%)	14 (28%)	7 (14%)
Patient's BMI >25kg/m2 (N=50)	3 (6%)	21 (42%)	22 (44%)	5 (10%)
Family History of CRC - Mother (N=50)	1 (2%)	13 (26%)	34 (68%)	3 (6%)
Family history of heart attack - mGFA (N=50)	1 (2%)	19 (38%)	28 (56%)	3 (6%)
OTHER FACTOR	2	0	0	0

References

1. Syngal S, Brand RE, Church JM, Giardiello FM, Hampel HL, Burt RW, et al. ACG clinical guideline: Genetic testing and management of hereditary gastrointestinal cancer syndromes. Am J Gastroenterol. 2015;110(2):223-62; quiz 63.
2. Stoffel EM, Mangu PB, Gruber SB, Hamilton SR, Kalady MF, Lau MW, et al. Hereditary colorectal cancer syndromes: American Society of Clinical Oncology Clinical Practice Guideline endorsement of the familial risk-colorectal cancer: European Society for Medical Oncology Clinical Practice Guidelines. J Clin Oncol. 2015;33(2):209-17.
3. Monahan KJ, Bradshaw N, Dolwani S, Desouza B, Dunlop MG, East JE, et al. Guidelines for the management of hereditary colorectal cancer from the British Society of Gastroenterology (BSG)/Association of Coloproctology of Great Britain and Ireland (ACPGBI)/United Kingdom Cancer Genetics Group (UKCGG). Gut. 2020;69(3):411-44.
4. Party. CCACCGW. Clinical practice guidelines for the prevention, early detection and management of colorectal cancer. Sydney: Cancer Council Australia 2017 [Available from: https://wiki.cancer.org.au/australiawiki/index.php?oldid=213460.
5. Vangala DB, Cauchin E, Balmana J, Wyrwicz L, van Cutsem E, Guller U, et al. Screening and surveillance in hereditary gastrointestinal cancers: Recommendations from the European Society of Digestive Oncology (ESDO) expert discussion at the 20th European Society for Medical Oncology (ESMO)/World Congress on Gastrointestinal Cancer, Barcelona, June 2018. Eur J Cancer. 2018;104:91-103.
6. Seppala TT, Latchford A, Negoi I, Sampaio Soares A, Jimenez-Rodriguez R, Sanchez-Guillen L, et al. European guidelines from the EHTG and ESCP for Lynch syndrome: an updated third edition of the Mallorca guidelines based on gene and gender. Br J Surg. 2021;108(5):484-98.
7. Network NCC. Genetic/Familial High-Risk Assessment: Colorectal. In: Network NCC, editor. NCNN Clinical Practice Guildeines in Oncology (NCCN Guidelines). USA: National Comprehensive Cancer Network; 2021.
8. Excellence NIfHaC. Colorectal Cancer. NICE guideline 2020 [Available from: https://www.nice.org.uk/guidance/ng151/resources/colorectal-cancer-pdf-66141835244485.
9. Giardiello FM, Allen JI, Axilbund JE, Boland CR, Burke CA, Burt RW, et al. Guidelines on genetic evaluation and management of Lynch syndrome: a consensus statement by the US Multi-Society Task Force on colorectal cancer. Gastroenterology. 2014;147(2):502-26.
